# Immersion-Driven Structural Evolution of NiFeS Nanosheets for Efficient Water Splitting

**DOI:** 10.3390/nano14010023

**Published:** 2023-12-20

**Authors:** Jianfeng Wang, Bingbing Zhao, Xiao Chen, Haixia Liu, Jie Zhang

**Affiliations:** 1 Key Laboratory for Liquid-Solid Structural Evolution and Processing of Materials, Ministry of Education, School of Materials Science and Engineering, Shandong University, Jingshi Road 17923, Jinan 250061, China; wangjianfeng@sdu.edu.cn; 2 Shandong Provincial Key Laboratory of Molecular Engineering, School of Chemistry and Chemical Engineering, Qilu University of Technology (Shandong Academy of Sciences), Jinan 250353, China; 17863659535@163.com (B.Z.); 10431220343@stu.qlu.edu.cn (X.C.); liuhaixia929@qlu.edu.cn (H.L.)

**Keywords:** immersion-sulfurization, sulfides, hydrogen evolution reaction, oxygen evolution reaction, overall water splitting

## Abstract

The development of low-cost, highly active, and stable electrocatalytic water-splitting catalysts is crucial to solving the current energy crisis and environmental pollution. Herein, a simple two-step conversion strategy is proposed to successfully prepare NiFeS nanosheet structure catalyst through the “immersion-sulfurization” strategy. The self-supported electrode can be prepared in large quantities due to its simple preparation process. As an active substance, NiFeS can grow directly on the NiFe foam substrate, avoiding the use of adhesives or conductive agents, and directly used as electrodes. The as-obtained NiFeS/NFF-300 displays efficient catalytic activity in electrocatalytic water splitting. The overpotential required for OER of the NiFeS/NFF-300 electrode at a current density of 10 mA cm^−2^ is 230 mV. The electrode underwent a stability test at 10 mA cm^−2^ for 24 h, and the overpotential remained essentially unchanged, demonstrating excellent stability. Moreover, NiFeS/NFF-300 exhibits considerable HER performances compared with NiFeC_2_O_4_/NFF and NiFe foam. The unique nanosheet structure and the presence of Ni^δ+^ and Ni^2+^ formed by NiFe foam substrate on the NiFeS surface are responsible for its excellent electrocatalytic activity.

## 1. Introduction

Global warming and the depletion of fossil fuels have prompted the exploration of renewable hydrogen energy, with electrocatalytic water splitting being an important way to obtain hydrogen energy [1,2,3]. The oxygen evolution reaction (OER) is a vital half-reaction for water splitting, which is restricted by the high activation energy, sluggish kinetics, and large overpotential of the four proton–electron transfer [4,5,6]. Developing efficient catalysts to optimize the OER process is crucial for hydrogen energy development. Currently, Ir- and Ru-based materials are state-of-the-art electrocatalysts for OER, but their high prices and scarcity hamper their large-scale applications [7,8,9].

Transition metal sulfides have emerged as a new class of water-splitting materials and have garnered significant attention and research [10,11,12]. Firstly, the good electronic transport properties and chemical reactivity are the key issues for excellent electrocatalytic activity, and transition metal sulfide materials can catalyze water electrolysis reactions at low potentials, enabling efficient hydrogen production. Additionally, compared with traditional Pt-based catalysts, transition metal sulfides offer better structural stability, allowing for more stable catalysis of hydrogen and oxygen generation during the electrolysis process, thereby significantly enhancing the efficiency of water splitting for hydrogen production [13]. Secondly, transition metal sulfides possess a tunable structure and composition. Sulfides with different structures and morphologies can be obtained by controlling the synthesis conditions, providing more possibilities for their application in water splitting and other energy conversion fields [14,15,16]. Moreover, transition metal sulfides exhibit the advantages of environmental friendliness and being easy to prepare. They can be produced and utilized through simple synthesis and regeneration processes. The waste products can be recycled, reducing environmental pollution and resource waste [17,18,19].

In recent years, a series of metal chalcogenide compounds such as MoS_2_ [20,21], NiFeS_x_ [22], CoS_2_ [23,24,25], and NiS_2_ [26,27,28] have been extensively studied for electrocatalytic water splitting. However, the OER activities fall short of expectations due to the slow transport kinetics and low activities on active sites in these materials. Therefore, there is an urgent need to develop new strategies to construct advanced materials with accelerated electrolyte diffusion pathways, high conductivity, sufficient active sites, and high intrinsic activity. NiFe bimetal-based sulfides exhibit enhanced OER kinetics in water splitting because of the synergistic electronic interaction between Ni and Fe [29,30]. Numerous studies show that the surface of NiFe-based sulfides would convert into their corresponding oxyhydroxides and oxides under the oxidation potential for the OER process [31,32]. The high conductivity and the doping effect of S into the in situ-formed hydroxides surface, which tunes the valence state of Ni/Fe, are responsible for their enhanced OER activities [33,34]. For example, Chen et al. fabricated Ni_x_Fe_1−x_S electrodes via electrodeposition-calcination processes, which present ultralow OER overpotential of 122 mV at 10 mA cm^−2^ in 1 M KOH [30]. DFT calculations reveal that the promoted OER energetics and enhanced electron localization function of Fe-O bonds on Fe_2_O_3_/FeOOH are responsible for the high activity of the Ni_x_Fe_1−x_S catalyst. Wang’s group constructed a sulfur-doped (Ni_7_Fe_3_)OOH-S OER electrode by facile sulfurization and anodic displacement processes [35]. The as-prepared electrode shows an overpotential of 238 mV at 10 mA cm^−2^ in 1 M KOH. DFT calculations reveal that the electronic structure and charge density of (NiFe)OOH were adjusted via the S dopant, thereby optimizing the OER rate-determining step and improving the binding energy for OER intermediates, which ensures high intrinsic catalytic activity.

Herein, a simple two-step transformation strategy was conducted to successfully prepare NiFeS nanosheet catalysts through an “immersion-sulfurization” approach. The self-supported electrode can be prepared in large quantities due to its simple fabrication process. Since the sulfides are grown directly on the NiFe foam, there is no need for binders or conductive additives, making it directly suitable for electrochemical testing. The NiFeS/NiFe electrode showed an overpotential of 230 mV for the oxygen evolution reaction (OER) at a current density of 10 mA cm^−2^. Moreover, the sample demonstrated excellent stability during a 24 h stability test at a current density of 10 mA cm^−2^, with minimal change in overpotential, indicating superior stability. Overall, this study presents a simple and scalable synthesis approach for NiFeS nanosheet catalysts using a soaking-sulfurization strategy. The resulting self-supported electrode exhibited excellent catalytic activity and stability for electrocatalytic water splitting, addressing the challenges associated with previous methods and offering new insights for the development of advanced materials in this field.

## 2. Experimental Section

### 2.1. Synthesis of NiFeC_2_O_4_/NFF

The NiFe foam (Ni:Fe = 1:1, NFF) and the S power were purchased from Kunshan Feimeite Electronic New Materials Co., Ltd., Kunshan, China and Tianjin Damao Chemical Reagent Co., Ltd., Tianjin, China, respectively. Ultrasonication in acetone (5 min) was performed for the removal of surface impurities from NFF, followed by rinsing with deionized water and ethanol to obtain a clean surface. Before immersion, the sample was placed in a hydrochloric acid solution (3 M) and sonicated for 10 min to obtain a rough surface. Then, the surface-roughened sample was immersed in a 0.5 M oxalic acid ethanol solution. The NFF area was 2 × 4 cm^2^, and the reaction was kept at a constant temperature of 45 ± 2 °C for 2 h. Afterward, the NFF was rinsed with deionized water and ethanol, drying in air completely. Thus, the precursor oxalic acid compound was received, marked as NiFeC_2_O_4_/NFF.

### 2.2. Synthesis of NiFeS/NFF

The as-synthesized NiFeC_2_O_4_/NFF (1 × 2 cm^2^) was placed at one side of the quartz boat, with the S powder (0.2 g) placed at the other side near the upstream of the airflow. Then, the quartz boat was placed at the center of the tube furnace, and the furnace was set to the desired temperature (250 °C) with a heating rate of 10 °C min^−1^ and remained at 250 °C for 2 h under Ar flow. Then, the furnace was turned off automatically and cooled down to room temperature naturally. Throughout the whole heat treatment process, the Ar gas provides stable gas protection and transports S vapor to the substrate to participate in the reaction with a flow rate of 20–30 sccm. Thus, the NiFeS at 250 °C supported on NFF was obtained, which was marked as NiFeS-250/NFF. In addition, heat treatment at 300 °C was also conducted for comparison to investigate the effect of temperature on the catalytic activities (NiFeS-300/NFF).

### 2.3. Material Characterizations

X-ray diffraction (XRD) results of samples were operated at an XD-3 diffractometer (Beijing Purkinje General Instrument Co., Ltd., Beijing, China) equipped with Cu Ka radiation. An energy-dispersive X-ray (EDX) analyzer-equipped scanning electron microscope (SEM, JSM-7610F, JEOL Ltd., Osaka, Japan) was utilized to characterize the microstructures and chemical compositions of the as-prepared samples. The transmission electron microscopy (TEM) and high-resolution TEM (HRTEM) measurements were conducted on a JEOL JEM 2100 field emission electron microscope (JEOL Ltd., Osaka, Japan). The selected area electron diffraction (SAED) patterns were also obtained to characterize the crystalline nature of the samples. Surface elemental information on samples was acquired by an X-ray photoelectron spectrometer (XPS, ESCALAB Xi+, Thermo Fisher, Cambridge, UK).

### 2.4. Electrochemical Measurements

Electrochemical measurements were conducted in a three-compartment electrochemical glass cell on a CHI760E (Chenhua, Shanghai, China). The graphite rod and Ag/AgCl (saturated KCl) electrode were utilized as counter and reference electrodes, respectively. The as-synthesized samples (NiFeS-250/NFF, NiFeS-300/NFF, NiFeC_2_O_4_/NFF, and NFF) were conducted as the working electrode directly. The potentials versus (vs.) Ag/AgCl were converted to the reversible hydrogen electrode (RHE) according to E_RHE_ = E_Ag/AgCl_ + 0.198 V + 0.059 pH. The linear sweep voltammetry (LSV) was conducted at a scan rate of 5 mV s^−1^ from 1 to 1.8 V vs. RHE in 1.0 M KOH. The fresh electrolytes were purged by high-purity O_2_ for 30 min before the OER measurement and throughout the whole test. Electrochemical impedance spectroscopy (EIS) measurements were performed at an excitation voltage of 5 mV with frequencies from 10^−2^ to 10^5^ Hz. To assess the electrochemically active surface areas (ECSAs), electrochemical double-layer capacitance (C_dl_) was performed using cyclic voltammetry (CV) technology in a non-Faradaic region (0.5–0.6 V vs. RHE) at different scan rates (10, 20, 30, 40, and 50 mV s^−1^). The OER durability was conducted by the chronopotentiometry (CP) at a constant current density of 10 mA cm^−2^ without *iR* compensation in 1.0 M KOH, while the LSV curves before and after durability tests were also acquired for comparison. All polarization curves were corrected via the equation *E_c_* = *E* − *iR_s_*, where *E_c_* is the *iR*-correction potential, *E* is the measured potential, and *R_s_* is the solution resistance obtained from EIS.

## 3. Results and Discussion

The NiFeS/NFF electrodes were acquired by the immersion-sulfurization strategy, and the optical photographs display the appearance and morphology of the sample at different stages (Figure S1). After the immersion of NFF in oxalic acid ethanol solution, the uniform NiFeC_2_O_4_ formed on the NFF surface.

**Figure 1:**
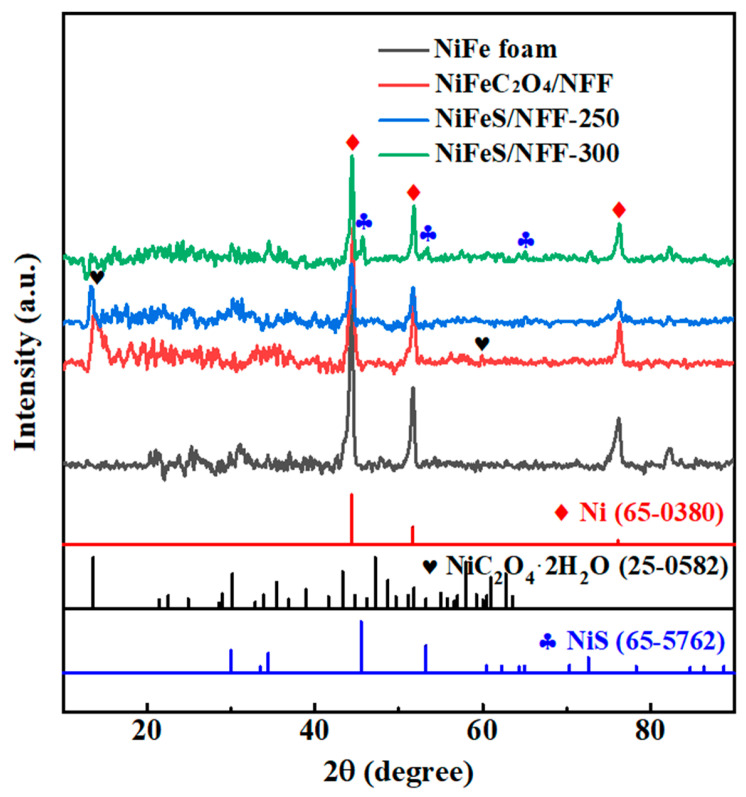
XRD patterns of different electrodes.

XRD analysis of NiFe foam, NiFeC_2_O_4_/NFF, and NiFeS/NFF samples was displayed in Figure 1. The presence of prominent peaks corresponding to NiC_2_O_4_·2H_2_O (JCPDS 25-0582) phase indicates the formation of NiFeC_2_O_4_ during the immersion process. After sulfurization, the NiS phase can be observed in NiFeS samples, which can be indexed to peaks at 45.5°, 53.3°, and 72.6°, corresponding to NiS (JCPDS 65-5762). Only the NiS phase was detected in the XRD pattern, suggesting that the Fe element in NiFeS is present in a solid solution form within the Ni lattice.

**Figure 2:**
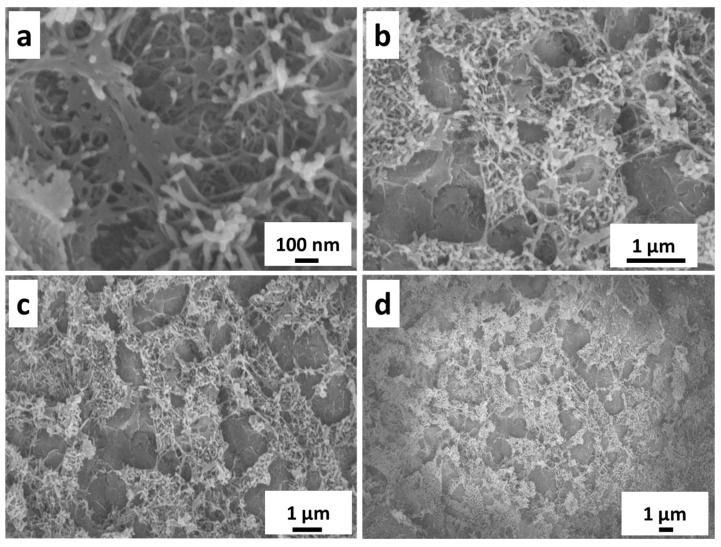
(**a**–**d**) SEM images of NiFeC_2_O_4_/NFF electrode at different magnifications.

**Figure 3:**
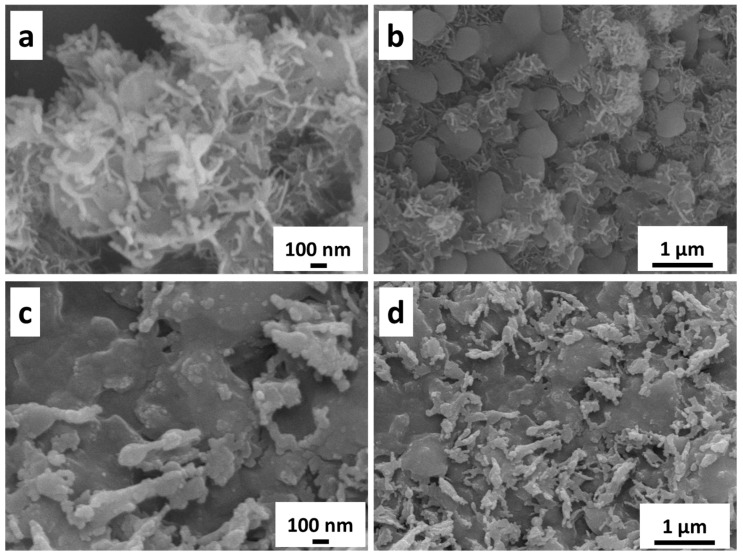
SEM images of (**a**,**b**) NiFeS/NFF-250 and (**c**,**d**) NiFeS/NFF-300 electrodes at different magnifications.

The SEM images showing the surface state of NFF are displayed in Figure 2, which presents the SEM images of NiFeC_2_O_4_ and reveals a mesh-like structure composed of intertwined nanowires, with the diameter of the nanowires being ~30 nm. The unique mesh-like structure greatly increases the specific surface area, laying a solid foundation for the enhanced catalytic activity of sulfide in subsequent processes. The sulfurization process converts oxalate compounds into sulfides, which exhibit significant changes in surface microstructure under SEM observation (Figure 3). Dispersed and uniform nanosheet structures are present on the surface, increasing the roughness of the sample surface. This may provide a larger surface area and more active sites for the electrocatalytic water-splitting reaction, enhancing the catalytic activity of the material and further reducing the overpotential and energy consumption during the water-splitting process.

**Figure 4:**
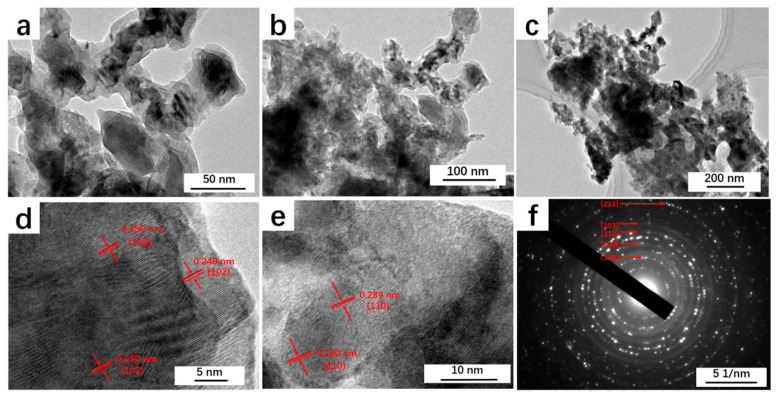
(**a**–**c**) TEM images, (**d**,**e**) HRTEM images, and (**f**) SAED pattern of NiFeS/NFF-300.

The TEM results of NiFeS/NFF-300 in Figure 4a–c display the nanosheet structure of NiFeS, consistent with the SEM results. HRTEM images clearly show lattice fringes (Figure 4d,e) with measured interplanar spacings of 0.25 nm and 0.29 nm, corresponding to the (102) and (101) crystal planes of NiS, respectively. To further confirm these lattice fringes, Figure 4f provides the corresponding selected-area electron diffraction (SAED) pattern, revealing the polycrystalline nature of the NiS nanosheets. The diffraction rings in the pattern correspond to the crystal planes of NiS at (100), (102), (110), (103), and (211), while no diffraction rings for Fe elements were observed. This further indicates that the Fe element in NiFeS exists in a solid solution form within the Ni lattice.

**Figure 5:**
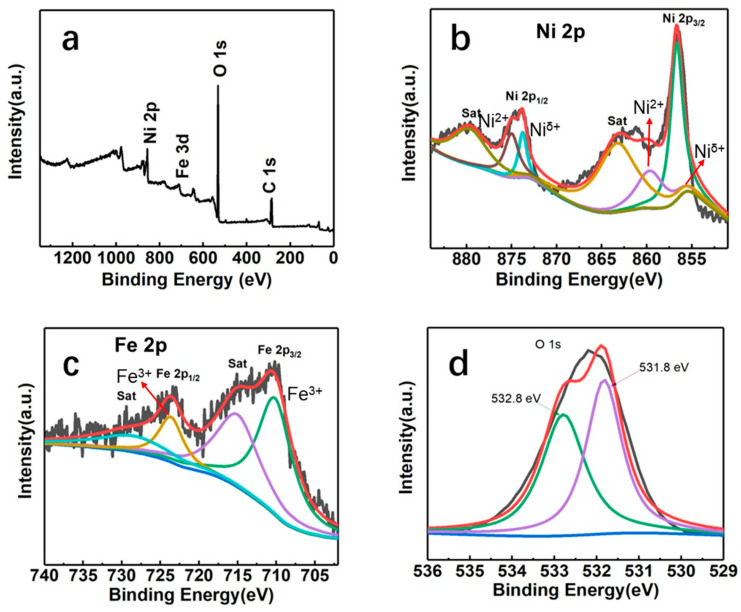
(**a**) XPS survey scan spectrum of NiFeC_2_O_4_/NFF, and the high-resolution XPS spectrum of (**b**) Ni 2p, (**c**) Fe 2p, and (**d**) O 1s regions of NiFeC_2_O_4_/NFF.

The composition of NiFeC_2_O_4_/NFF was investigated using XPS measurements. As shown in Figure 5a, the presence of peaks corresponding to Ni, Fe, C, and O confirms the existence of these four elements. Figure 5b presents the Ni 2p orbital spectra. The peaks located at 856.7 and 859.7 eV in the Ni 2p_3/2_ orbital indicate the presence of Ni^δ+^ and Ni^2+^ in the NiFeC_2_O_4_/NFF. Similarly, the peaks corresponding to Ni^δ+^ and Ni^2+^ in the Ni 2p_1/2_ orbital are observed at 873.8 and 879.7 eV, respectively. The satellite peaks associated with the Ni 2p_3/2_ and Ni 2p_1/2_ orbitals are observed at 863.2 and 889.9 eV, respectively. Figure 5c shows the Fe 2p spectrum. The peaks at 723.8 and 710.4 eV are attributed to Fe^3+^ in Fe_2_O_3_ corresponding to the Fe 2p_1/2_ and Fe 2p_3/2_ orbitals, respectively, while the peaks at 715.4 and 728.8 eV are related to satellites of Fe 2p_1/2_ and Fe 2p_3/2_ orbitals, respectively. The O 1s spectrum is displayed in Figure 5d, from which the peaks at 531.8 and 532.8 eV can be observed. The oxygen vacancies and adsorbed molecular water are responsible for the occurrence of these two peaks.

**Figure 6:**
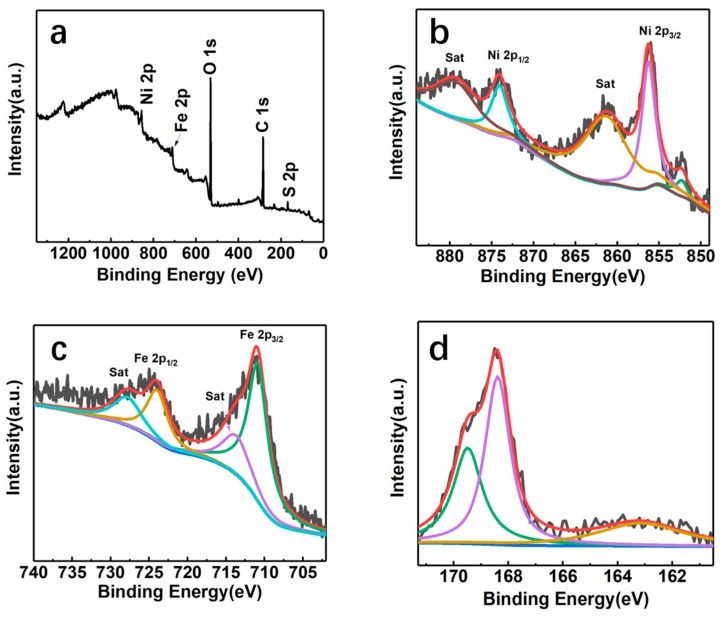
(**a**) XPS survey scan spectrum of NiFeS/NFF-250, and the high-resolution XPS spectrum of (**b**) Ni 2p, (**c**) Fe 2p, and (**d**) S 2p regions of NiFeS/NFF-250.

**Figure 7:**
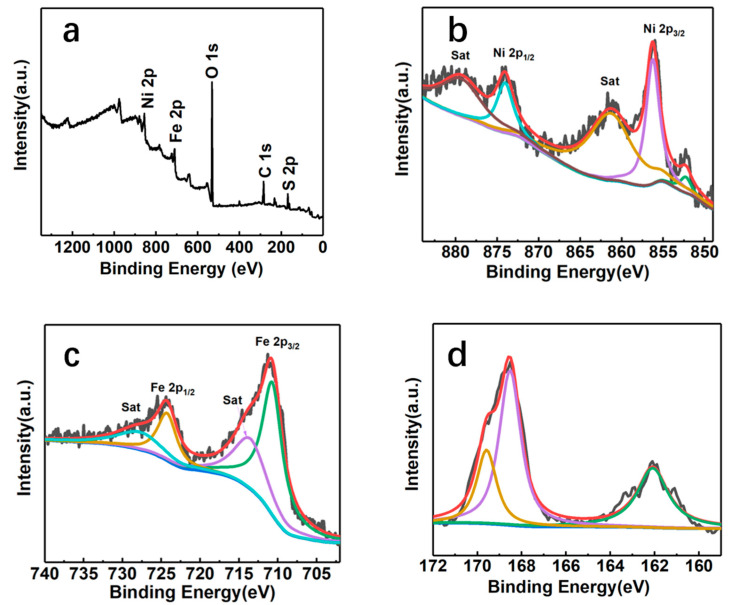
(**a**) XPS survey scan spectrum of NiFeS/NFF-300, and the high-resolution XPS spectrum of (**b**) Ni 2p, (**c**) Fe 2p, and (**d**) S 2p regions of NiFeS/NFF-300.

The XPS survey spectrum of the NiFeS/NFF electrode at 250 °C is shown in Figure 6a, which confirms the presence of Ni, Fe, S, O, and C elements on the electrode surface. Figure 6b presents the Ni 2p spectrum of NiFeS/NFF-250. In the 2p_3/2_ orbital, the peak at 852.4 eV is attributed to the oxidation of Ni in the NiFe foam, forming Ni^δ+^ and exhibiting a 0.2 eV shift compared to the literature value of metallic Ni at 852.6 eV. The peak at 855.5 eV is attributed to the oxidation state of Ni^2+^ on the surface of the generated NiFeS material. From the Ni 2p_1/2_ orbital, the peak at 874.1 eV corresponds to Ni^2+^. The peaks at 861.5 and 879.8 eV are attributed to the satellite peaks of Ni 2p_3/2_ and Ni 2p_1/2_ orbitals, respectively. Figure 6c displays the XPS spectrum of Fe 2p, showing peaks at 724.1 eV for Fe 2p_1/2_ and 711.0 eV for Fe 2p_3/2_. The shift of the Fe^3+^ peak from 717.7 to 724.1 eV indicates a strong electronic interaction between S and Fe, leading to charge redistribution on S and Fe. The satellite peaks at 714.4 and 728.0 eV correspond to the Fe 2p_3/2_ and Fe 2p_1/2_ orbitals, respectively, indicating the predominant presence of Fe^3+^ oxidation state in NiFeS. In Figure 6d, the high-resolution S spectrum exhibits two prominent peaks at 168.4 and 169.5 eV, corresponding to the oxidized states of sulfur in nickel-iron sulfide. Figure 7 shows the XPS spectrum of the NiFeS electrode synthesized at 300 °C. It can be observed that the intensity of peaks corresponding to the S element has significantly increased compared with that synthesized at 250 °C, indicating a higher S content in the NiFeS electrode at 300 °C. The peaks corresponding to other elements show no changes in intensity and position compared with that at 250 °C, suggesting no other chemical transformations occurred.

**Figure 8:**
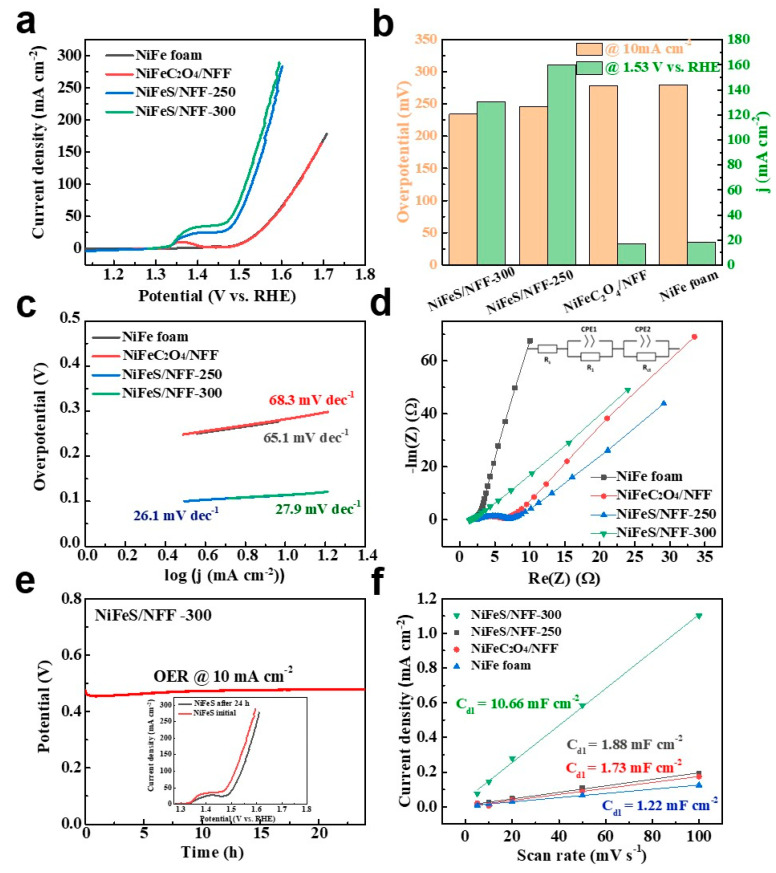
Electrocatalytic performances of different catalysts in 1 M KOH for OER. (**a**) LSV polarization curves, (**b**) comparison of overpotentials at 10 mA cm^−2^ and current densities at the overpotential of 300 mV, (**c**) Tafel plots, (**d**) Nyquist plots at an overpotential of 270 mV, (**e**) chronopotentiometry curves of NiFeS/NFF-300 at 10 mA cm^−2^ (the insets illustrate LSV polarization curves before and after the stability test), and (**f**) the capacitive currents at 0.65 V vs. RHE as a function of scan rates for different catalysts.

The sulfides can form strong chemical bonds with transition metals, thereby enhancing their stability [36]. NiFeS/NFF electrodes were synthesized through immersion of NiFeC_2_O_4_/NFF electrodes followed by sulfurization. The unique three-dimensional network structure grew on the skeleton surface of the Ni foam (Figure 2). The NiFeC_2_O_4_ nanostructures were further transformed into NiFeS nanosheets during the subsequent vulcanization treatment. Optical photographs of NiFe foam, NiFeC_2_O_4_/NFF, and NiFeS/NFF are shown in Figure S1. To evaluate the OER performance of these catalysts, NiFeS/NFF, NiFeC_2_O_4_/NFF, and NiFe foam electrodes were tested in a 1.0 M KOH solution. Figure 8a shows that the NiFeS/NFF-250 and NiFeS/NFF-300 electrodes exhibited excellent OER catalytic activities compared with NiFeC_2_O_4_/NFF and NiFe foam. In particular, NiFeS/NFF and NiFeC_2_O_4_/NFF electrodes already exhibited an oxidation peak before the onset of OER, indicating the occurrence of oxidation reactions, corresponding to the transition from Ni^2+^ to Ni^3+^ [37,38,39]. It can be observed that the NiFeC_2_O_4_/NFF and NFF electrodes exhibited similar overpotentials of 270 and 273 mV, respectively, at a current density of 10 mA cm^−2^. In contrast, the NiFeS/NFF electrodes achieved a current density of 10 mA cm^−2^ at an overpotential of 230 mV. Due to the occurrence of oxidation reactions before OER, the polarization curves exhibited a large oxidation peak, resulting in an overall elevation of the polarization curve during OER, making it difficult to precisely determine the overpotential at a current density of 10 mA cm^−2^. Therefore, the overpotentials at a current density of 100 mA cm^−2^ were observed, with values of 284, 307, and 307 mV for NiFeS/NFF, NiFeC_2_O_4_/NFF, and NFF electrodes, respectively, indicating the superior OER performance of NiFeS/NFF. Figure 8b compares the important parameters for OER performance evaluation, such as the overpotential (η) at the current density of 10 mA cm^−2^, and the current densities (*j*) at the overpotential of 300 mV of these four electrodes, from which the OER activity of the four electrodes is clear at a glance. Table S1 compares the overpotentials at 10 mA cm^−2^ between the NiFeS/NFF electrode with other non-precious OER electrocatalysts, demonstrating that the NiFeS/NFF electrode has lower overpotential than most catalysts, indicating the excellent electrocatalytic performance for water splitting. Figure 8c shows the Tafel slopes obtained from the polarization curves of the electrodes. The Tafel slope of NiFeS/NFF electrode (27.9 mV dec^−1^) was significantly lower than that of NiFeC_2_O_4_/NFF (82.4 mV dec^−1^) and NiFe foam (65.1 mV dec^−1^). A smaller Tafel slope indicates faster kinetics and a lower overpotential at the same current, implying that the NiFeS/NFF electrode exhibits higher OER activity compared with NiFeC_2_O_4_/NFF and NiFe foam. Figure 8d presents the EIS results of the electrodes at 1.5 V (vs. RHE). A smaller EIS value indicates lower charge transfer resistance, and the order of R_ct_ values for the three electrodes is NiFeS/NFF (2.8 Ω) > NiFeC_2_O_4_/NFF (4.5 Ω) > NiFe foam (>100 Ω) [40]. The EIS further elucidates the higher catalytic activity of the NiFeS/NFF electrode. The electrochemical stability of NiFeS/NFF was tested, and it exhibited good stability even after 24 h (Figure 8e). Based on the inset in Figure 8e, it can be observed that after 24 h of durability measurement, there is a slight increase in overpotential at a current density of 10 mA cm^−2^. Visual inspection of the sample surface reveals the presence of a reddish-brown substance, suggesting the formation of iron rust resulting from oxidation of the sample. SEM images of NiFeS/NFF-300 after the OER durability at different magnifications are shown in Figure S2. The nanosheets exhibit a certain degree of aggregation but still maintain good integrity. The electrochemically active surface areas (ECSAs) of the electrodes were calculated from double-layer capacitance (C_dl_), acquired from cyclic voltammetry in a potential range free of Faradaic processes [41,42]. Figure S3a–d shows the CV curves conducted at 0.6–0.7 V (vs. RHE) at different scan rates from 20 to 120 mV s^−1^. As shown in Figure 8f, the C_dl_ value of NiFeS/NFF-300 is calculated to be 10.66 mF cm^−2^, which is much larger than that of NiFeS/NFF-250 (1.88 mF cm^−2^), NiFeC_2_O_4_/NFF (1.73 mF cm^−2^), and NiFe foam (1.22 mF cm^−2^). The general application of C_dl_ in a water-splitting study is to calculate the ECSA by employing the following relation:

$$\mathrm{ECSA}=\mathrm{Geometrical}\;\mathrm{Surface}\;\mathrm{area}\;\times \;\frac{C_{dl}}{C_{s}}$$

where C_s_ represents the capacitance of the flat electrode surface (C_s_ = 0.04 mF cm^−2^). So, the calculated ECSA values for NiFeS/NFF-300, NiFeS/NFF-250, NiFeC_2_O_4_/NFF, and NiFe foam were 266.5, 47, 43.25, and 30.5 cm^2^, respectively. Such a large ECSA of NiFeS/NFF-300 indicates more available active sites, which explains the enhanced OER performances and is consistent with the rough surface in SEM images.

**Figure 9:**
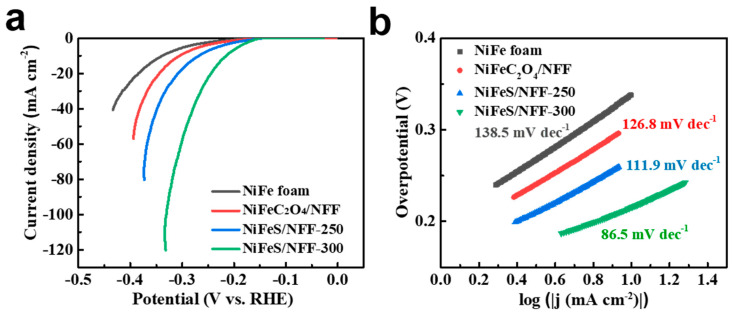
HER performances of different electrodes in 1 M KOH. (**a**) HER polarization curves, and (**b**) corresponding Tafel plots.

The HER activity of different electrodes was evaluated. The *iR*-corrected LSV curves of different catalysts at 5 mV s^−1^ are displayed in Figure 9a, in which NiFeS/NFF-300 exhibits the smallest overpotential at 10 mA cm^−2^ (215 mV). The overpotential required for NiFeS/NFF-250 at 10 mA cm^−2^ is 268 mV, which is smaller than that of NiFeC_2_O_4_/NFF (305 mV) and NiFe foam (338 mV). The resulting Tafel slope of NiFeS/NFF-300 is 86.5 mV dec^−1^ (Figure 9b), which is smaller than those of NiFeS/NFF-250 (111.9 mV dec^−1^), NiFeC_2_O_4_/NFF (126.8 mV dec^−1^), and NiFe foam (138.5 mV dec^−1^).

The NiFeS/NFF electrode exhibits excellent electrocatalytic activity for water splitting, which can be attributed to several factors. Firstly, NiFeS is formed directly on the NFF surface through the immersion method, where NiFeC_2_O_4_·2H_2_O intermediate is synthesized and then sulfurized to obtain NiFeS. The in situ growth of NiFeS on the NFF allows for direct contact between NiFeS and the substrate, improving the overall conductivity of the electrode. Additionally, the NiFeS/NFF electrode requires no binders or conductive additives, enabling it to be directly used as a working electrode. Secondly, the electrocatalytic activity can be evaluated based on charge transfer capability. EIS results reveal that the NiFeS/NFF electrode exhibits the smallest R_ct_ value compared with the others, indicating faster charge transfer kinetics. The rapid charge transfer rate on the electrode surface reduced the possibilities of the catalytic reaction being limited by charge transfer. Furthermore, the uniform distribution of NiFeS nanosheets facilitates the transport of electrolytes and relevant ions. The large surface area of the NiFeS nanostructures enhances the catalytic activity of the reaction. The two-dimensional nanosheet structure of NiFeS provides a larger surface area, offering more active sites for the OER and thus improving the catalytic performance. In summary, the NiFeS/NFF electrode possesses high conductivity and fast charge transfer kinetics, along with a large electrochemically active surface area and numerous catalytic active sites. These factors collectively contribute to the excellent electrocatalytic activity of the electrode for water electrolysis.

## 4. Conclusions

NiFeS nanomaterials were prepared by the “immersion-sulfurization” method. The preparation of the intermediate product NiFeC_2_O_4_·2H_2_O provides a morphological basis for the subsequent preparation of sulfides. The linear network nanostructures and two-dimensional nanosheet structures can be observed in NiFeC_2_O_4_/NFF and NiFeS/NFF electrodes, respectively. In the electrocatalytic water splitting measurement in the KOH electrolyte, the NiFeS/NiFe electrode showed excellent electrocatalytic performance, with an OER voltage of 1.47 V at a current density of 10 mA cm^−2^. The following factors contribute to the excellent electrocatalytic activity of NiFeS/NFF electrodes. Firstly, the in situ growth of the NiFeS active substance on NFF needs no binders or conductive agents, and can be used as an electrode for electrocatalytic measurement directly. Secondly, the smallest R_ct_ value of the NiFeS/NFF electrode indicates the fastest charge transfer dynamics. Thirdly, the large specific surface area caused by the distribution of a large number of nanosheets will increase the electrochemically active area, which can provide more active sites for HER and OER. Finally, the presence of Ni^δ+^ and Ni^2+^ formed by NiFe foam substrate on the NiFeS surface is also one important reason for its excellent electrocatalytic activity. These factors work together to provide excellent electrocatalytic activity for NiFeS/NFF electrodes, and their development in the field of electrocatalytic water splitting is worth looking forward to.

## Data Availability

Data are contained within the article and Supplementary Materials.

## Supplementary Materials

The following supporting information can be downloaded at: https://www.mdpi.com/article/10.3390/nano14010023/s1, Figure S1. Optical photographs showing the fabrication processes of the NiFeS/NFF-250 and NiFeS/NFF-300 electrodes. Figure S2. SEM images of NiFeS/NFF-300 after the OER durability at different magnifications. Figure S3. CVs for (a) NiFeS/NFF-300, (b) NiFeS/NFF-250, (c) NiFeC_2_O_4_/NFF, and (d) NiFe foam measured in the potential range from 0.6 to 0.7 V vs. RHE at different scan rates from 5 to 100 mV s^−1^ in the 1 M KOH. Table S1. Comparison of the electrocatalytic OER performance of the NiFeS with other non-precious. References [43,44,45,46,47,48,49,50,51,52,53,54,55,56,57] are cited in the supplementary materials.
