# Supplementary material for: Immersion-Driven Structural Evolution of NiFeS Nanosheets for Efficient Water Splitting
Source: Nanomaterials (Basel). 2023 Dec 20;14(1):23. doi: 10.3390/nano14010023 (PMC10780408; doi:10.3390/nano14010023)
Supplement: Supplementary file 1 [file nanomaterials-14-00023-s001.zip › nanomaterials-2744932-supplementary.pdf]

Supporting Information

Immersion-Driven Structural Evolution of NiFeS Nanosheets for Efficient Water Splitting

Jianfeng Wang [1], Bingbing Zhao [2], Xiao Chen [2], Haixia Liu [2] and Jie Zhang [2,*]

[1] Key Laboratory for Liquid-Solid Structural Evolution and Processing of Materials, Ministry of Education, School of Materials Science and Engineering, Shandong University, Jingshi Road 17923, Jinan 250061, China; wangjianfeng@sdu.edu.cn
[2] Shandong Provincial Key Laboratory of Molecular Engineering, School of Chemistry and Chemical Engineering, Qilu University of Technology (Shandong Academy of Sciences), Jinan 250353, China; 17863659535@163.com (B.Z.); 10431220343@stu.qlu.edu.cn (X.C.); liuhaixia929@qlu.edu.cn (H.L.)
* Correspondence: zh_jie@qlu.edu.cn

This part includes:

Figure S1-S3

Table S1

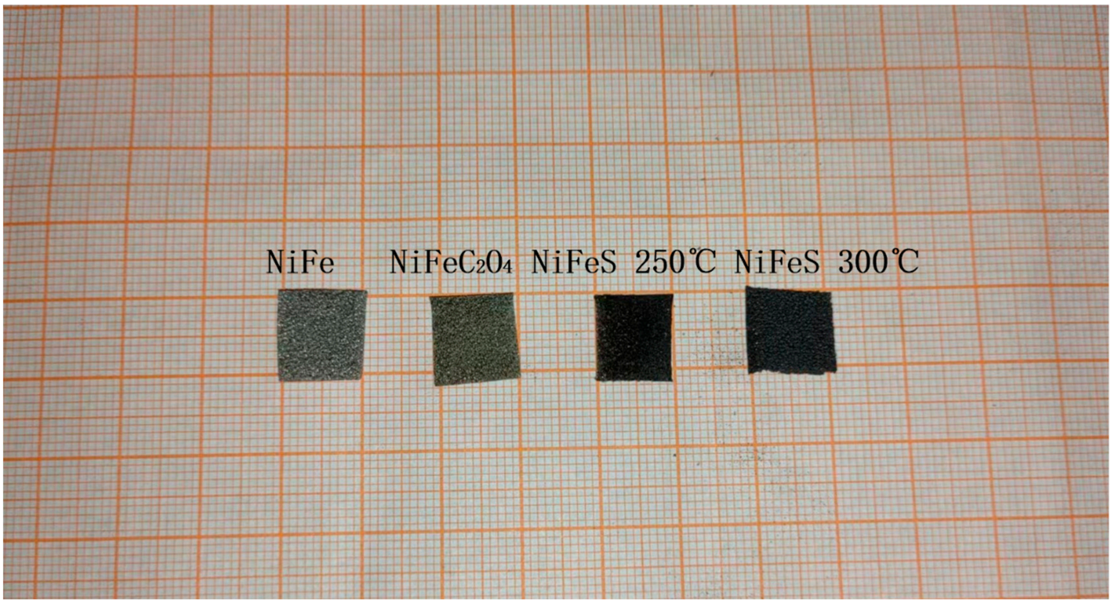

Figure S1. Optical photographs showing the fabrication processes of the NiFeS/NFF-250 and NiFeS/NFF-300 electrodes.

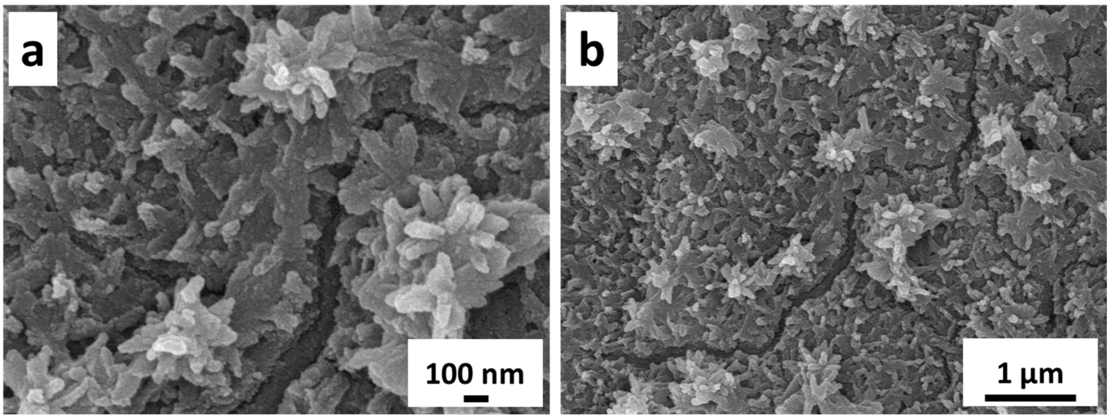

Figure S2. SEM images of NiFeS/NFF-300 after the OER durability at different magnifications.

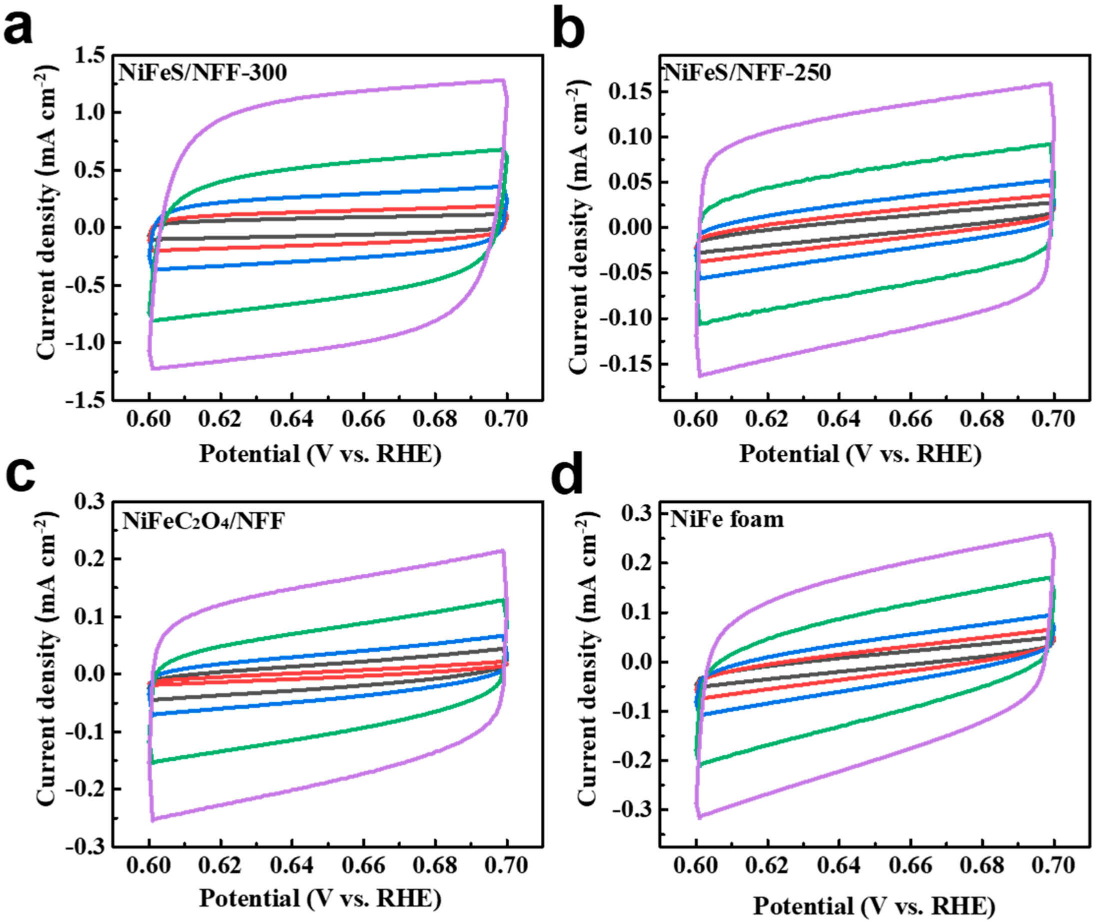

Figure S3. CVs for (a) NiFeS/NFF-300, (b) NiFeS/NFF-250, (c) $NiFeC_2O_4$/NFF, and (d) NiFe foam measured in the potential range from 0.6 to 0.7 V vs. RHE at different scan rates from 5 to 100 mV s^{-1} in the 1 M KOH.

Table S1. Comparison of the electrocatalytic OER performance of the NiFeS with other non-precious

catalysts	η_{10} (mV)	Reference
$CoFe_2O_4$ NSs	275	[1]
NiFeHCH	389	[2]
MoS_2@CoO	325	[3]
α-Mn_2O_3	340	[4]
Co_6W_6C@NC	268	[5]
CeO_x CoP	264	[6]
$Ni_{0.33}Co_{0.67}S_2$	320	[7]
Co_3O_4@PPy	440	[8]
$CoSe_2$ CNTs	300	[9]
$CuCo_2S_4$ CNS	269	[10]
VFe-MOF@NF	246	[11]
$FeNi_3$@NC	277	[12]
Ni-Fe-P-B	269	[13]
NiS_2/MoS_2	234	[14]
Ni/$NiSP_x$	231	[15]
**NiFeS/NFF**	**230**	**Our work**

References

1. Fang, H.; Huang, T.; Liang, D.; Qiu, M.; Sun, Y.; Yao, S.; Yu, J.; Dinesh, M. M.; Guo, Z.; Xia, Y.; Mao, S. Prussian blue analog-derived 2D ultrathin $CoFe_2O_4$ nanosheets as high-activity electrocatalysts for the oxygen evolution reaction in alkaline and neutral media. *J. Mater. Chem. A* **2019,** *7* (13), 7328-7332.

2. Karthick, K.; Anantharaj, S.; Ede, S. R.; Kundu, S. Nanosheets of nickel iron hydroxy carbonate hydrate with pronounced OER activity under alkaline and near-neutral conditions. *Inorg. Chem.* **2019,** *58* (3), 1895-1904.

3. Cheng, P.; Yuan, C.; Zhou, Q.; Hu, X.; Li, J.; Lin, X.; Wang, X.; Jin, M.; Shui, L.; Gao, X.; Nötzel, R.; Zhou, G.; Zhang, Z.; Liu, J. Core-shell MoS_2@CoO electrocatalyst for water splitting in neural and alkaline solutions. *J. Phys. Chem. C* **2019,** *123* (10), 5833-5839.

4. Kölbach, M.; Fiechter, S.; van de Krol, R.; Bogdanoff, P. Evaluation of electrodeposited α-Mn_2O_3 as a catalyst for the oxygen evolution reaction. *Catal. Today* **2017,** *290*, 2-9.

5. Chen, J.; Ren, B.; Cui, H.; Wang, C. Constructing pure phase tungsten-based bimetallic carbide nanosheet as an efficient bifunctional electrocatalyst for overall water splitting. *Small* **2020,** *16* (23), e1907556.

6. Zhang, T.; Wu, X.; Fan, Y.; Shan, C.; Wang, B.; Xu, H.; Tang, Y. Hollow CeOx/CoP heterostructures using two-dimensional Co-MOF as template for efficient and stable electrocatalytic water splitting. *ChemNanoMat* **2020,** *6* (7), 1119-1126.

7. Peng, Z.; Jia, D.; Al-Enizi, A. M.; Elzatahry, A. A.; Zheng, G. From water oxidation to reduction: homologous Ni-Co based nanowires as complementary water splitting electrocatalysts. *Adv. Energy Mater.* **2015,** *5* (9), 1402031.

8. Tong, Y.; Liu, H.; Dai, M.; Xiao, L.; Wu, X. Metal-organic framework derived Co_3O_4/PPy bifunctional electrocatalysts for efficient overall water splitting. *Chinese Chem. Lett.* **2020,** *31* (9), 2295-2299.

9. Wei, G.; Du, K.; Zhao, X.; Wang, J.; Yan, W.; An, C.; An, C. Cable-like carbon nanotubes decorated metal-organic framework derived ultrathin $CoSe_2$/CNTs nanosheets for electrocatalytic overall water splitting. *Chinese Chem. Lett.* **2020,** *31* (10), 2641-2644.

10. Hao, Z.; Wei, P.; Yang, Y.; Sun, J.; Song, Y.; Guo, D.; Liu, L. Self-assembled $CuCo_2S_4$

nanosheets with rich surface Co^{3+} as efficient electrocatalysts for oxygen evolution reaction. *Appl. Surf. Sci.* **2021,** *536,* 147826.

11. Han, L.; Xu, J.; Huang, Y.; Dong, W.; Jia, X. High-performance electrocatalyst of vanadium-iron bimetal organic framework arrays on nickel foam for overall water splitting. *Chinese Chem. Lett.* **2021,** *32* (7), 2263-2268.

12. Chen, D.; Zhu, J.; Mu, X.; Cheng, R.; Li, W.; Liu, S.; Pu, Z.; Lin, C.; Mu, S. Nitrogen-doped carbon coupled $FeNi_3$ intermetallic compound as advanced bifunctional electrocatalyst for OER, ORR and Zn-air batteries. *Appl. Catal. B: Environ.* **2020,** *268,* 118729.

13. Tang, W.; Liu, X.; Li, Y.; Pu, Y.; Lu, Y.; Song, Z.; Wang, Q.; Yu, R.; Shui, J. Boosting electrocatalytic water splitting via metal-metalloid combined modulation in quaternary Ni-Fe-P-B amorphous compound. *Nano Res.* **2020,** *13* (2), 447-454.

14. Li, C.; Liu, M.; Ding, H.; He, L.; Wang, E.; Wang, B.; Fan, S.; Liu, K. A lightly Fe-doped (NiS_2/MoS_2)/carbon nanotube hybrid electrocatalyst film with laser-drilled micropores for stabilized overall water splitting and pH-universal hydrogen evolution reaction. *J. Mater. Chem. A* **2020,** *8* (34), 17527-17536.

15. Chen, X.; Li, Q.; Che, Q.; Chen, Y.; Tan, Y.; Xu, X. Self-supported Ni/$NiSP_x$ microdendrite structure for highly efficient and stable overall water splitting in simulated industrial environment. *ACS Sustain. Chem. Eng.* **2019,** *7* (13), 11778-11786.
